# Phenylacetic acid mediates Acinetobacter baumannii entry into a viable but non-culturable state

**DOI:** 10.1099/mic.0.001650

**Published:** 2026-01-16

**Authors:** Lyuboslava G. Harkova, Rubén de Dios, Ronan R. McCarthy

**Affiliations:** 1 Antimicrobial Innovations Centre, Department of Life Sciences, College of Health and Life Sciences, Brunel University London, Uxbridge, UB8 3PH, UK; 2 National Biofilm Innovation Centre, School of Biological Sciences, Faculty of Environmental and Life Sciences, University of Southampton, University Road, Southampton, SO17 1BJ, UK

**Keywords:** *Acinetobacter baumannii*, biofilm, desiccation, phenylacetic acid, viable but non-culturable

## Abstract

Desiccation tolerance is central to the pathogenic success of the opportunistic pathogen *Acinetobacter baumannii*, allowing its survival on hospital surfaces in the absence of water and nutrients for months at a time, compromising surface decontamination and aiding cross-contamination between staff and patients. Despite the importance of desiccation tolerance, the regulation underpinning this behaviour remains largely elusive. In this work, transcriptomic analyses of desiccated cells revealed phenylacetic acid (PAA) catabolism as an essential mediator of desiccation tolerance. We subsequently demonstrate that deletion of the *paa* operon abolished the clonogenicity of desiccated cells. Strikingly, these *A. baumannii* cells remained viable by entering the viable but non-culturable (VBNC) state, a means to survive extreme stressors like antibiotic exposure. Furthermore, we uncover that PAA catabolism is necessary to mediate PAA-driven biofilm regulation. These findings highlight PAA catabolism as a modulator of biofilm formation and a key pathway for entry into the VBNC state in response to desiccation. This reveals PAA catabolism as a target for novel infection prevention strategies.

## Data Availability

RNA-seq data have been deposited at the National Centre for Biotechnology Information Gene Expression Omnibus public database with the accession number GSE229096.

## Introduction

*Acinetobacter baumannii* is a nosocomial pathogen highly proficient at colonizing surfaces in healthcare settings. These colonized surfaces can act as *A. baumannii* reservoirs, leading to outbreaks associated with high costs, prolonged patient hospitalization and reduced quality of life [1,2]. A major determinant of *A. baumannii*’s persistence on surfaces is its outstanding ability to survive long-term desiccation [3,4]. A range of environmental and intrinsic factors impact this behaviour, including relative humidity, temperature, nutrients and oxygen availability, growth phase and cell size [5,9]. Additional contributors to this recalcitrant behaviour are lipid A acetylation, capsule production, compatible solutes and protein aggregation prevention resulting from the protective function of DtpA and DtpB hydrophilins [5,10,17]. There is a growing body of evidence showing that *A. baumannii* enters a viable but non-culturable (VBNC) state when impacted by water-limited conditions and that this is a key mediator of its capacity for prolonged desiccation tolerance [18,19]. VBNC cells have increased tolerance to antimicrobials and retain their virulence [18,19] while also leading to false assessment of successful decontamination procedures due to the lack of culturability under standard laboratory conditions. Thus, *A. baumannii* VBNC cells pose a great risk of persistence and dissemination of this pathogen in healthcare settings. However, the regulatory cues governing *A. baumannii*’s entry into the VNBC state during desiccation remain elusive.

In this study, we assessed the global transcriptional response to desiccation in nutrient-limiting conditions in an effort to recreate the nosocomial desiccation scenario. Analyses of these datasets uncovered the phenylacetic acid (PAA) catabolic pathway as a regulator of VBNC state under desiccation in this pathogen, with the genes encoding this pathway being the most upregulated in these conditions. We subsequently demonstrate that a Δ*paa* operon deletion mutant, which accumulates PAA, is entirely non-culturable after 72-h desiccation but critically remains as viable as the WT strain. Taken together, this work establishes a regulatory link between the PAA catabolic pathway, the VBNC state and desiccation tolerance in *A. baumannii*.

## Methods

### Bacterial strains and growth conditions

A mixed culture of both phase variants of *A. baumannii* AB5075 [20] strain was used for assessing the transcriptome of desiccated and non-desiccated cells and opaque variants of the WT AB5075 and the Δ*paa* mutant were used for the subsequent assays (Table S3, available in the online Supplementary Material). Strains were grown in M9 minimal medium supplemented with sodium succinate (40 mM), lysogeny broth (LB) or their solid versions with 15 g l^−1^ agar. Host *Escherichia coli* strains were routinely grown in LB broth or agar.

### Mutant strain construction

For constructing a Δ*paa* clean deletion mutant in the *paa* operon, we used the strategy described by de Dios *et al*. [21]. Initially, 1 kb homologous regions upstream and downstream of the *paa* operon were amplified from AB5075 genomic DNA using paaOpr up fw/rv and paaOpr down fw/rv primer pairs, respectively (Table S3). The two fragments were joined together by overlapping PCR and cloned in SmaI-digested pEMGT vector, resulting in pEMGT-paa derivative.

To construct the Δ*paa* mutant, the pEMGT-paa plasmid was transferred to AB5075 by triparental mating using pRK2013 as helper and a first recombination event was selected on LB agar with ampicillin (100 µg ml^−1^) and tellurite (30 µg ml^−1^) and verified by PCR using Tel fw/rv oligos pair. A second recombination was triggered by transferring the pSW-Apr plasmid to the AB5075/pEMGT-paa cointegrate strain and selecting on LB agar with ampicillin (100 µg ml^−1^) and apramycin (200 µg ml^−1^). The deletion of the *paa* operon was validated by PCR using paaOpr up fw/down rv oligos. The pSW-Apr was removed from the final Δ*paa* mutant by serial passaging.

To fluorescently label the WT AB5075 and its derivative Δ*paa* mutant, strains carrying a miniTn7-Tc-mChartreuse insertion were constructed as described by Ducas-Mowchun *et al*. [22,23]. First, we modified the pUC18T-miniTn7T-Gm plasmid [24] (Addgene, #63121) to be used according to the antibiotic resistance profile of AB5075. For this, the tetracycline resistance cassette from pUC18T-miniTn7T-Tc-lacI^q^-Ptac [25] was amplified by PCR using the primer pair tetA fw/tetA rv [21] and cloned in pUC18T-miniTn7T-Gm cut with EagI and BsrGI and blunted with Klenow. This resulted in pUC18T-miniTn7T-Tc. After this, we cloned the coding sequence of the fluorescent protein mChartreuse constitutively expressed from a non-repressed *Ptac* promoter. The mChartreuse coding sequence was amplified from plasmid pNF02-mChartreuse [23] using primers Ptac RBS pNF02 fw HindIII, which included the *Ptac* promoter and a ribosome binding site, and pNF02 rv KpnI. The resulting PCR product was digested with HindIII and KpnI and cloned in pUC18T-miniTn7T-Tc digested with the same enzymes, generating pUC18T-miniTn7T-Tc-Ptac::mChartreuse.

The miniTn7T-Tc-Ptac::mChartreuse construct was inserted in the *att*Tn7 site of the WT AB5075 and its derivative Δ*paa* mutant through four-parental mating, as described by Ducas-Mowchun *et al*. [22], using pRK2013 and pTNS2 as helper plasmids [26,27]. Transconjugants were selected on LB agar supplemented with tetracycline (5 mg l^−1^) and chloramphenicol (15 mg l^−1^). The resulting fluorescently labelled strains were validated by PCR using primers AB5075 glmS fw and Tn7R [21].

Plasmids and oligonucleotides used in this work are listed in Table S3. All constructs were validated by Sanger sequencing.

### RNA extraction and sequencing

Bacterial cultures grown in M9-succinate were washed three times with distilled water and then split in two. Each bacterial suspension was equalized to OD_600_=3 in distilled water or M9-succinate (1 : 250, v:v dilution). Cells were harvested from 1 ml of the bacterial suspension adjusted in water and resuspended in RNA*later*™ for RNA integrity preservation, subsequently used as control non-desiccated cells. Five hundred microlitres of each OD-adjusted sample was dropped on Petri dish lids and aseptically air-dried. All samples were desiccated in a closed chamber for 24 h at room temperature (19±2 °C) and 10±2% relative humidity, maintained with Drierite desiccant. Cells were rehydrated directly in 1.5 ml RNA*later*™, harvested by gentle scraping and pipetting and stored at −80 °C until RNA extraction. The experiment was done in three independent biological replicates.

Total RNA from each sample was isolated using Qiagen RNAeasy Kit according to the manufacturer’s instructions. Samples were treated with on-column DNase digestion (Qiagen) and additionally treated with RNase-free DNase (Invitrogen). RNA quality was checked using Bioanalyzer. The library was prepared with Illumina Stranded Total RNA Prep Ligation with Ribo-Zero Plus kit and 10 bp IDT for Illumina indices. NovaSeq 6000 was used for the sequencing, giving 2×51 bp reads. Differentially expressed genes were defined by log_2_ (fold change) ≥ 1 or ≤ −1 and significance (*P* value) < 0.05. Gene set enrichment analysis (GSEA) was performed using FUNAGE-Pro with default settings [28]. Data was represented in volcano plots using VolcaNoseR [29]. The resulting transcriptomic datasets are available at the Gene Expression Omnibus repository (NCBI) with accession number GSE229096.

### Growth curves and biofilm assay

Bacterial growth and biofilm formation were measured as previously described [30]. Cultures were grown overnight (~18 h) in LB broth or M9-succinate at 37 °C 180 r.p.m. OD_600_ was measured and adjusted to 0.1 in each corresponding medium. For continuous growth assessment, 200 µl of OD-adjusted culture was transferred per well in a standard 96-well plate and absorbance was measured at 600 nm (OD_600_) every 30 min over a 16-h period at 37 °C 200 r.p.m. Bacterial growth was measured by the area under the curve [31] calculated using GraphPad Prism. For the biofilm assay, 150 µl of each adjusted culture was used to inoculate the corresponding wells of a 96-well plate. Following 24-h growth at 37 °C 180 r.p.m., all biofilms were washed three times with distilled water, stained with 200 µl 0.1% Crystal violet for 15 min and washed five times to remove excess stain. All biofilms were then air-dried and the Crystal violet was resolubilized in 99% ethanol for at least 3 h. Absorbance was measured after that at 570 nm (OD_570_).

### Desiccation and viable/culturable cell quantification assay

The desiccation tolerance of *A. baumannii* AB5075 and the Δ*paa* mutant was tested as previously described [32] with slight modifications. Overnight cultures of the fluorescently labelled WT AB5075/miniTn7-Tc-mChartreuse and Δ*paa*/miniTn7-Tc-mChartreuse mutant were grown in M9-succinate. Bacterial cells from 1 ml of each overnight culture were harvested by centrifugation and washed twice with distilled water. All bacterial suspensions were adjusted to OD_600_=1 in water. Five microlitres were diluted in 20 µl sterile PBS, 10 µl of which were used to determine culturable cells by serial dilutions, spot plating on M9-succinate agar and overnight incubation at 37 °C, while the remaining 10 µl was diluted in 500 µl PBS and used to determine the viable cell count using flow cytometry. Five microlitres of OD-adjusted samples was pipetted on a plastic surface, aseptically air-dried and desiccated in a closed chamber in the dark at 5.7±1.5% relative humidity and 21±0.5 °C ambient temperature. Samples were rehydrated for 5 min in 20 µl PBS. Half of this volume was used for determining culturable cells and half of the volume was used for determining the viable cells as described above.

To determine cell viability using fluorescence as a proxy, samples were run in an ACEA Novocyte Flow Cytometer 3000 (Agilent Technologies). The event detection threshold was set to 2000 on FSC (forward scatter). The mChartreuse fluorescent signal was detected through the FITC channel (excitation/emission: 495/519 nm). Fluorescent events in 100 µl of the 500 µl cell suspensions indicated above were measured to determine viable cells.

### Data analysis

Each assay was performed in biological triplicate. Statistical analyses were performed using GraphPad Prism (v10.4.2, San Diego, CA, USA, https://www.graphpad.com/) and are specified in the figure legends.

## Results

### *A. baumannii* upregulates the PAA catabolic pathway under desiccation

**Fig. 1.**
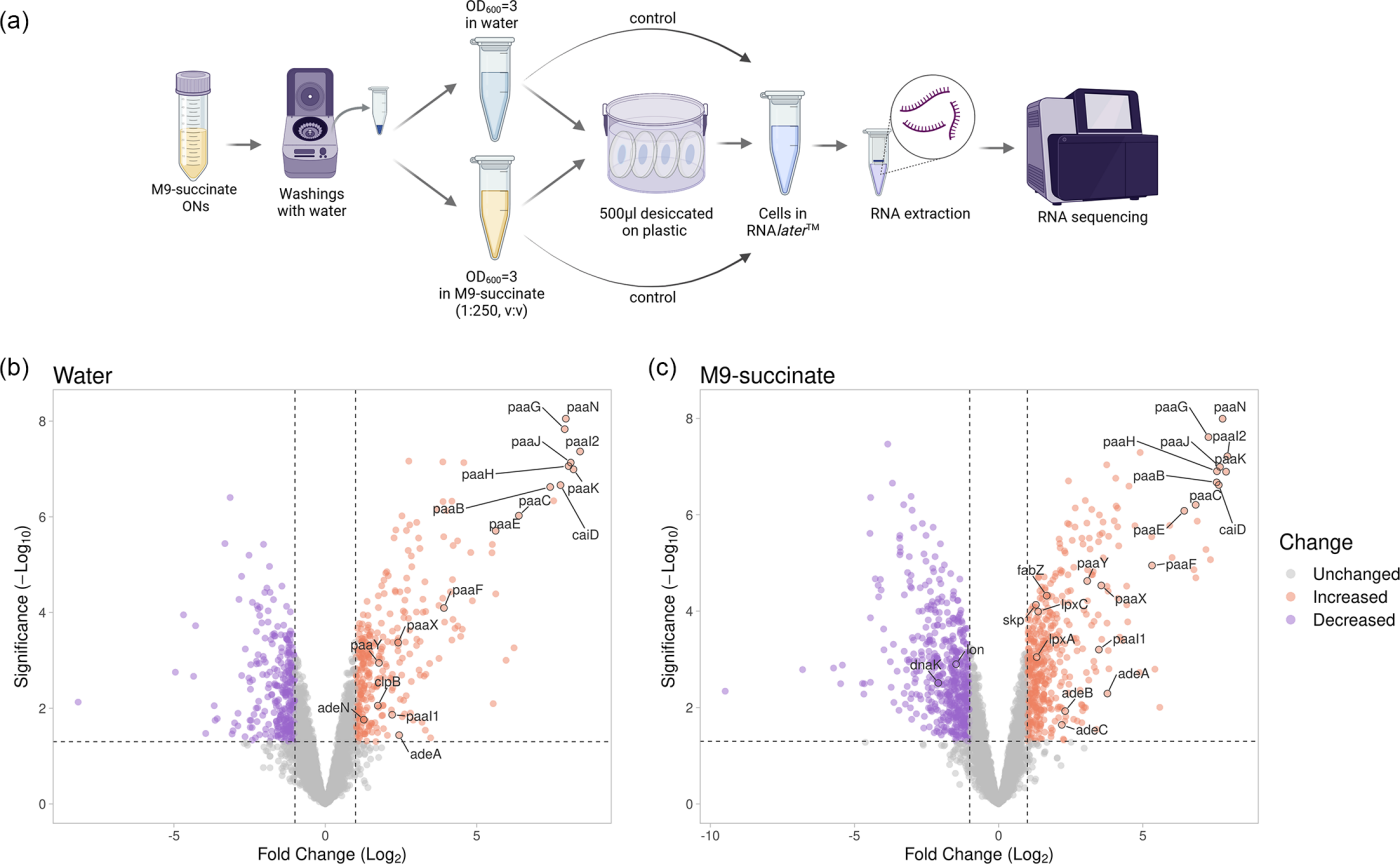
PAA catabolism is related to *A. baumannii* desiccation tolerance. (a) Experimental set-up of sample preparation for RNA extraction and sequencing. Overnight (ON) cultures grown in M9-succinate were desiccated in water or diluted M9-succinate (1:250, v:v) for 24 h at 10±2% relative humidity and ambient temperature (17–18 °C). Image was created using Biorender.com. (b, c) Volcano plots presenting dRNA-seq data of the transcriptomes of cells desiccated in water (b) and diluted M9-succinate (c). (b) Five hundred eighty-eight genes were differentially expressed in the water desiccated cells, of which 279 were downregulated (purple dots) and 309 were upregulated (orange dots). Highlighted are the upregulated genes related to PAA catabolism (*paa* operon), protein stabilization (*clpB*) and RND efflux pumps (*adeA* and *adeN*). (c) Nine hundred forty-seven genes were differentially expressed when cells were desiccated in diluted M9-succinate, 512 of which were down- and 435 were upregulated. Downregulated highlighted genes are linked to protein aggregation prevention (*lon* and *dnaK*), while the upregulated genes are involved in PAA catabolism (*paa* operon), outer membrane protein stabilization and lipid A production (*skp*, *fabZ*, *lpxA* and *lpxC*) and RND efflux pump (*adeABC*).

To understand the global gene expression changes during the adaptation of *A. baumannii* AB5075 to desiccation, we performed differential RNA sequencing (dRNA-seq) comparing *A. baumannii* cells suspended either in water or diluted M9-succinate minimal medium and desiccated for 24 h to cells sampled before desiccation (Fig. 1a). We selected these conditions to more accurately recapitulate desiccation on fomites in a nosocomial environment. We decided to include cells suspended in diluted M9-succinate in the experiment to differentiate between genes regulated by desiccation and genes regulated by the lack of nutrients while avoiding the precipitation of media components during the drying process. As a result, 588 and 947 genes were differentially expressed comparing cells desiccated in water and cells desiccated in diluted M9-succinate, respectively, to the control (Fig. 1b, c). Five hundred sixty-nine genes were differentially expressed between the samples desiccated in water and diluted minimal medium (Table S1).

In agreement with previous studies [14], our transcriptomic experiment showed a downregulation of *lon* and *dnaK*, which prevent protein aggregation, in cells desiccated in diluted M9-succinate (Fig. 1c). Furthermore, cells desiccated in water and in diluted M9-succinate showed an upregulation of the chaperones *clpB* and *skp*, respectively (Fig. 1c). *skp* is involved in the production of lipid A, which is crucial for *A. baumannii* desiccation tolerance [12]. This aligns with our results, which show an upregulation of the lipid A biosynthetic genes *fabZ*, *lpxA* and *lpxC* (log_2_(FC)=1.7, 1.3 and 1.4, respectively). Consistent with previous findings [12], we saw upregulation of RND efflux pumps encoding genes *adeA* and *adeN* in water-dried cells (log_2_(FC)=2.4, 1.2, 1.3, respectively), as well as *adeABC* (log_2_(FC)=3.8, 2.3 and 2.2, respectively) in diluted M9-succinate desiccated cells (Fig. 1c).

There was one pathway, the PAA catabolic pathway (gene organization shown in Fig. S1), whose expression was consistently upregulated in both conditions, indicative of a desiccation-specific response. In water-desiccated cells, *paaB*, *caiD*, *paaH*, *paaK*, *paaG*, *paaN*, *paaJ* and *paaI2* were upregulated by >7-log_2_(FC) compared to non-desiccated cells. *paaE* and *paaC* expression was increased by >6 fold, while *paaF, paaI1*, *paaY* and *paaX* were upregulated by 3.9, 2.2, 1.8 and 2.4-log_2_(FC), respectively, compared to control cells (Fig. 1b). Similarly, in the cells desiccated in diluted M9-succinate, *paaB*, *caiD*, *paaJ*, *paaK*, *paaI2*, *paaN*, *paaG* and *paaH* were upregulated >7-log_2_(FC) compared to non-desiccated cells, while the other *paa* operon genes *paaC*, *paaE*, *paaF*, *paaX*, *paaI1* and *paaY* were upregulated by 7, 6, 5, 3.6, 3.5 and 3-log_2_(FC), respectively (Fig. 1c).

To further assess gene expression trends for each comparison, we performed a GSEA. The results showed that, among the upregulated pathways, the PAA catabolic pathway was the most enriched in cells desiccated in water or diluted M9-succinate compared to non-desiccated cells (Table S2). Meanwhile, fatty acid catabolism and nucleotide biosynthesis were the most enriched pathways among the significantly downregulated genes in both desiccation conditions (Table S2). Altogether, our transcriptomic results suggest that low levels of PAA are required for the early adaptation to desiccation, linking the PAA catabolic pathway and this recalcitrant *A. baumannii* behaviour.

### PAA accumulation augments biofilm formation despite abrogating desiccation tolerance

**Fig. 2.**
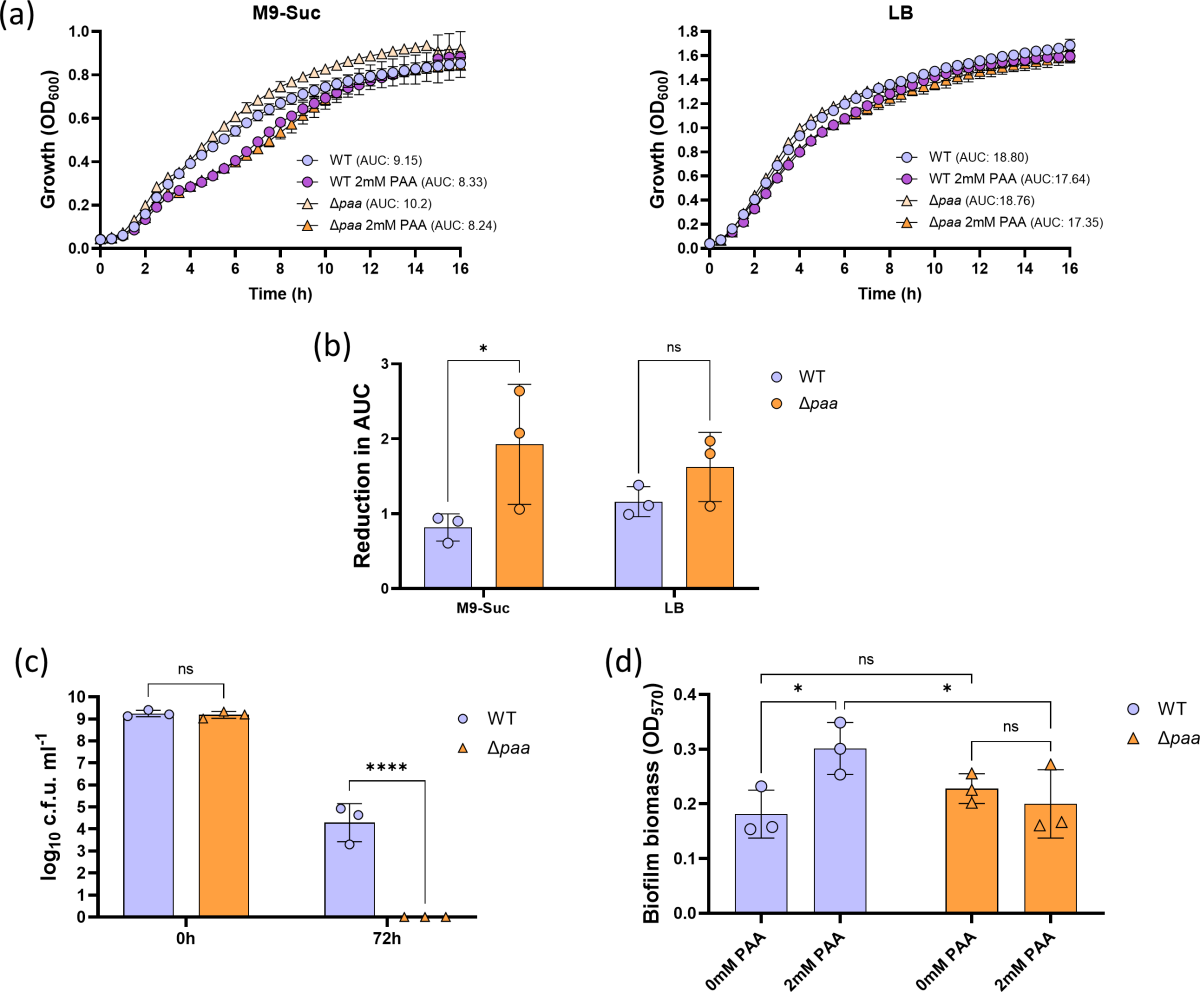
PAA accumulation affects *A. baumannii* fitness, desiccation and biofilm formation. (a, b) Growth curves of *A. baumannii* AB5075 WT and Δ*paa* mutant in M9-succinate (M9-Suc) or LB broth supplemented with exogenous 2 mM PAA. Overnight cultures in M9-Suc or LB broth were adjusted to OD_600_ 0.1 and grown at 37 °C shaking. Continuous growth was assessed by measuring absorbance at 600 nm (OD_600_) every 30 min for 16 h, the AUC was calculated for each sample (a) and the reduction in the AUC following exposure to exogenous PAA was calculated (b). (c) Desiccation tolerance of WT and Δ*paa* mutant determined by the number of c.f.u. after 72-h desiccation at 19.5±1.5 °C and 8.5±3% RH. (d) Biofilms of the WT and Δ*paa*, grown in LB broth for 24 h, were stained with 0.1% Crystal violet which was subsequently resolubilized in 99% ethanol. Absorbance was measured at 570 nm (OD_570_). ns *P*>0.05, *P<0.05, ***P<0.001 (Two-Way ANOVA with Šídák post-hoc test).

To explore the potential role of PAA catabolism in the physiology of *A. baumannii* AB5075, we constructed a clean-deletion mutant in the *paa* operon (Δ*paa*). Mutations in this catabolic pathway lead to PAA accumulation [33]. To assess if the accumulation of PAA directly affects *A. baumannii* fitness and if this could influence culturability, we compared the growth of the Δ*paa* mutant to that of the WT AB5075 in M9-succinate and LB (Fig. 2a). In M9-succinate, the Δ*paa* mutant showed a mildly higher growth rate than the WT, with areas under the curve (AUC) of 10.2 and 9.15, respectively. In contrast, both strains grew similarly in LB (AUCs of 18.80 and 18.76, respectively). To further potentiate the effect of PAA accumulation, we repeated the growth experiments supplementing the media with PAA 2 mM (Fig. 2a). Whereas PAA did not show a substantial impact on the growth of both the WT and the Δ*paa* mutant in LB, it produced a negative impact on their growth in minimal medium (AUCs of 8.33 and 8.24, respectively). This supports the idea that PAA has a greater effect on the physiology of *A. baumannii* under nutrient-limiting conditions.

To assess the effect of PAA catabolism on *A. baumannii* desiccation, we performed a standard desiccation assay [7,9,34] where cells of the Δ*paa* mutant were kept in dry conditions (8.5±3% relative humidity) over 72 h before being rehydrated and assessed based on their clonogenicity. Our results showed that the absence of the *paa* operon abolished clonogenicity of *A. baumannii* after 72-h desiccation (Fig. 2c). This indicates that PAA accumulation negatively regulates desiccation tolerance in this pathogen.

Currently, the established paradigm is that biofilm formation is positively associated with desiccation tolerance [15]. Thus, we hypothesized that the observed effect of PAA accumulation on decreased desiccation tolerance could be associated with impaired biofilm formation. To test this for AB5075, we measured the biofilm formation of the WT AB5075 and its Δ*paa* mutant derivative in LB medium (M9-succinate medium supports a negligible amount of biofilm formation in our experimental conditions, as shown in Fig. S2). Deletion of the *paa* operon did not significantly alter the biofilm biomass compared to the WT (Fig. 2d). As with the growth assays, to further potentiate the effect of PAA accumulation, we repeated the assays supplementing with 2 mM PAA. *A. baumannii* AB5075 formed significantly more biofilm in LB supplemented with PAA 2 mM (Fig. 2d). However, this effect of PAA supplementation on biofilm formation was abolished in the Δ*paa* mutant, indicating that PAA catabolization is needed to enhance biofilm formation. Altogether, our results challenge the established paradigm positively linking biofilm levels and desiccation tolerance.

### PAA regulates VBNC state of desiccated cells in *A. baumannii*

**Fig. 3.**
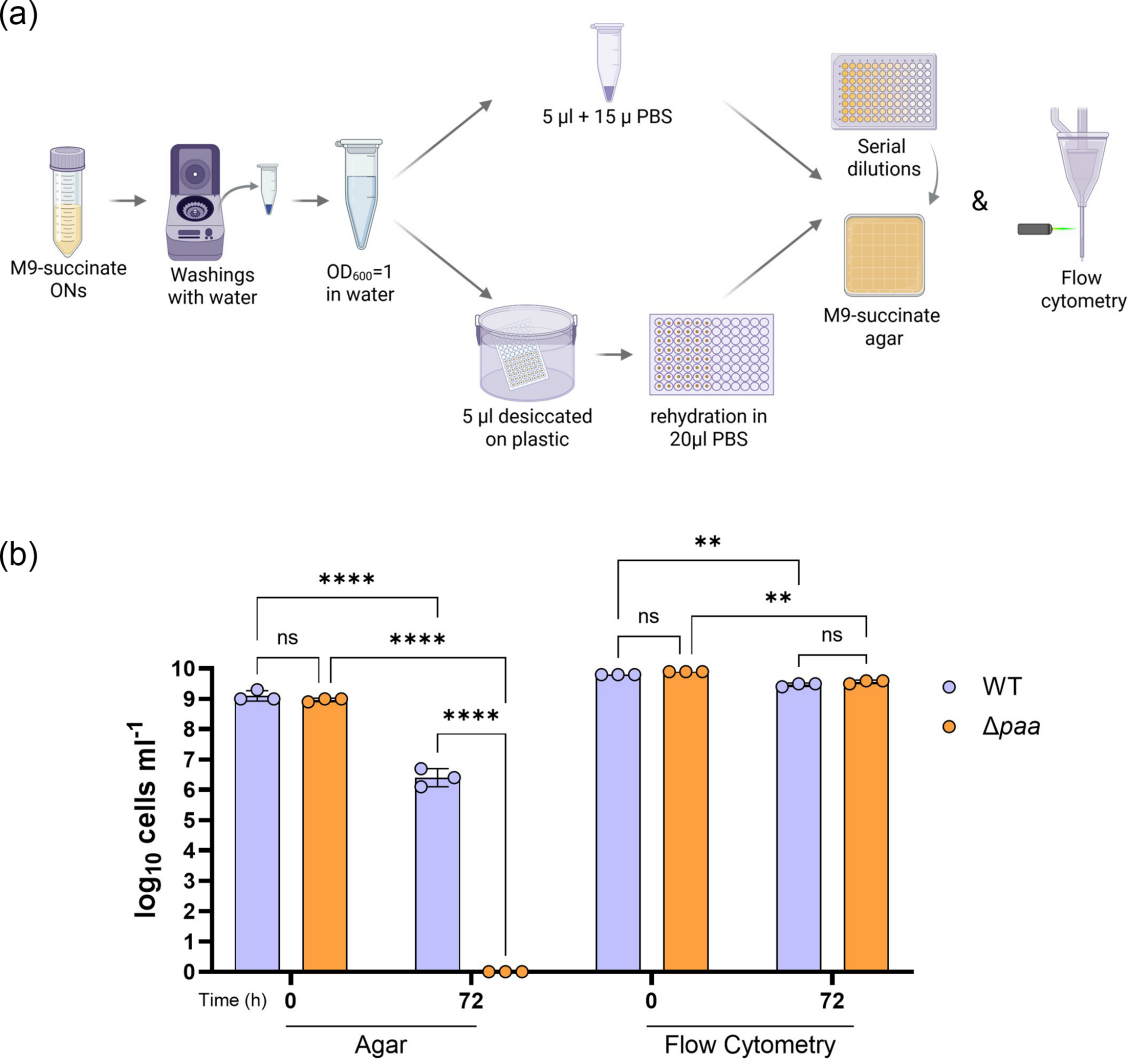
PAA accumulation drives *A. baumannii* in a non-culturable state under desiccation. (a) Experimental set-up of the desiccation assay using culturing and flow cytometry to determine viable cells before and after desiccation of fluorescently labelled AB5075. Image was created using Biorender.com. (b) Effect of PAA catabolism on *A. baumannii* cells determined by the number of culturable (agar) and viable (flow cytometry) fluorescently labelled WT and Δ*paa* mutant before (0 h) and after (72 h) desiccation at controlled humidity (5.7±1.5%) and ambient temperature (21±0.5 °C). The experiment was done in triplicates; data represent mean±sd (represented by error bars). ns *P*>0.05, *P<0.05, **P<0.01, ***P<0.001, ****p<0.0001 (Two-Way ANOVA with Šídák post-hoc test).

Our data suggested that deletion of the *paa* operon abolished desiccation tolerance after 72 h (Fig. 2), but surprisingly, this was not related to decreased biofilm formation (Fig. 2). It was recently shown that the desiccation process can cause a proportion of the cell population to switch to the VBNC state [18,19]. Furthermore, VBNC cells (non-clonogenic) retain fluorescence conferred by the production of a fluorescent protein as opposed to dead cells [19]. To test if PAA accumulation affects desiccation-mediated entrance in VBNC state in *A. baumannii*, we generated fluorescently labelled derivatives of the WT AB5075 and the Δ*paa* mutant constitutively expressing the fluorescent protein mChartreuse. Then, we grew both fluorescently labelled strains in M9-succinate before being desiccated. The same samples were subjected to culture-based c.f.u. counting and flow cytometry (using fluorescence as a proxy for cell viability) before and after 72-h desiccation [5,7, 8, 13, 19]. Strikingly, the direct detection of fluorescently labelled viable cells showed no significant difference in the cell counts between the WT and Δ*paa* mutant, before and after desiccation, whereas the culturability of the Δ*paa* mutant was abolished after desiccation (Fig. 3). Thus, our results demonstrate that, under nutrient-limiting conditions, PAA accumulation drives *A. baumannii* cells into the VBNC state during desiccation.

## Discussion

PAA is an emerging stress and virulence signalling molecule in *A. baumannii* [33,35, 36]. It is involved in bacterial resistance to antibiotics, biofilm regulation and osmotic pressure, as well as in the immunomodulation of neutrophils. Additionally, the PAA catabolic pathway, which controls the levels of PAA, appears differentially regulated in *A. baumannii* during intramacrophage colonization [37]. Our transcriptomics data showed that the *paa* operon is the most significantly upregulated group of genes in the clinical isolate AB5075 during desiccation under nutrient-limiting conditions (Fig. 1). The established paradigm positively associates desiccation tolerance with biofilm levels, and previous studies have demonstrated that PAA accumulation promotes biofilm formation [15,33]. Interestingly, while we validated the previously shown increase in biofilm formation in response to PAA exposure [33,34], our data demonstrates that active PAA catabolization is required for this effect (Fig. 2). This suggests that the molecule involved in modulating *A. baumannii* biofilm formation is a metabolite generated during PAA breakdown, rather than the PAA itself. Moreover, we show that the absence of PAA degradation reduces culturability during desiccation despite not affecting biofilm formation (Figs2  3), which challenges the current paradigm.

**Fig. 4.**
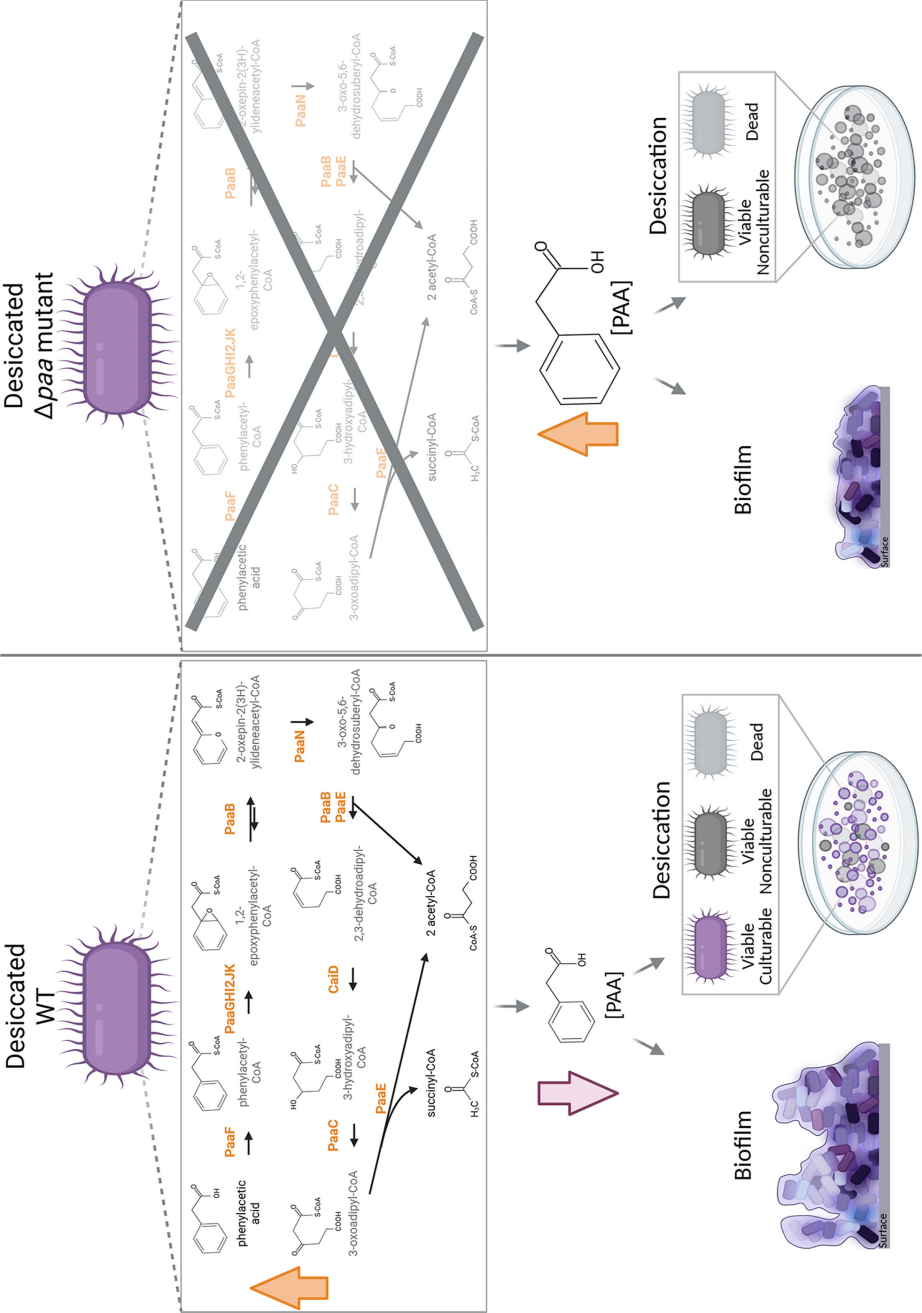
Role of PAA catabolism in *A. baumannii* AB5075 biofilm formation and desiccation tolerance. Dried WT cells increase the expression of the *paa* operon, which enhances PAA degradation and lowers PAA concentrations, resulting in a dried population of predominantly viable and culturable cells. In contrast, the absence of the *paa* operon (Δ*paa*) and the catabolic pathway leads to increased PAA levels in the cells, which decreases biofilm formation and shifts the desiccated population to predominantly VBNC cells. Created in BioRender.com.

The presence of VBNC cells during desiccation prompts a shift in the assessment of *A. baumannii*’s capability to withstand the pressures of desiccation, which has thus far been based on the ability of viable cells to grow on laboratory media [7,9,34]. A recent report showed that exogenous PAA increases the recovery of culturable *A. baumannii* MCC 2076 cells post-desiccation after being grown in rich media initially [34], which highlights the importance of nutrient availability when assessing desiccation tolerance. Intriguingly, another recent study established a link between desiccation tolerances and VBNCs in *A. baumannii*, highlighting that a proportion of cells lose culturability upon air-drying [19]. Building on these compelling findings, we validated the role of PAA degradation during desiccation by testing viability and culturability before and after desiccation of a fluorescently labelled Δ*paa* compared to the WT AB5075. This showed a direct link between the absence of the PAA catabolic pathway, and hence the accumulation of PAA, and entrance into the VBNC state of *A. baumannii* under desiccation. The mutation of the PAA catabolic pathway led to loss of culturability but not viability of AB5075, grown under nutrient-limiting conditions, thereby prompting entry into the VBNC state during desiccation (Fig. 4). This is particularly important as bacteria experience nutrient limitations by the human host [38] and patients are a major source of hospital surface contamination with pathogenic bacteria [1]. Thus, it is likely that *A. baumannii* cells persisting on hospital surfaces experience nutrient limitations both before and during desiccation. An important protective strategy in both desiccated and VBNC cells is preventing protein aggregation [14,39]. Hence, it is plausible that PAA catabolism is related to the regulation of DtpA and DtpB hydrophilins, preventing the aggregation of proteins in desiccated VBNC *A. baumannii* cells. Importantly, upon rehydration and resuscitation in biological buffers and human biological fluids, the desiccated VBNC *A. baumannii* cells have been shown to retain virulence [19]. Although multiple *A. baumannii* strains have shown the ability to enter into a VBNC state, their capacity to do so varies depending on the stressor and happens in a strain-dependent manner [18]. Hence, based on our results linking VBNC state and desiccation tolerance via PAA regulation, it is possible that the difference among *A. baumannii* strains in their desiccation tolerance reported in the literature [3] could be due to variations in their capacity to enter into the VBNC state. Consequently, this could be attributed to strain-specific differences in intracellular PAA levels. Future work will focus on deleting the *paa* operon in different *A. baumannii* clinical isolates and evaluating the intracellular PAA concentrations, desiccation tolerance and VBNC entry during desiccation among mutants and the WT parental strains of these isolates. Uncovering the role of PAA as a mediator of VBNC state during desiccation also highlights a key metabolic vulnerability that could be targeted in future disinfection treatments and outbreak prevention strategies. This highlights the need for DNA-based detection methods, such as viable quantitative PCR (vqPCR) [40], which have improved VBNC accuracy for better detection of pathogens like the critically important pathogen *A. baumannii*.

## Supplementary material

10.1099/mic.0.001650 Uncited Supplementary Material 1.

10.1099/mic.0.001650 Uncited Table S1.

10.1099/mic.0.001650 Uncited Table S2.

10.1099/mic.0.001650 Uncited Table S3.

## Abbreviations

AUC: area under the curve
dRNA-seq: differential RNA sequencing
FSC: forward scatter
GSEA: gene set enrichment analysis
LB: lysogeny broth
PAA: phenylacetic acid
VBNC: viable but non-culturable
