# Supplementary material for: Phenylacetic acid mediates Acinetobacter baumannii entry into a viable but non-culturable state
Source: Microbiology (Reading). 2026 Jan 16;172(1):001650. doi: 10.1099/mic.0.001650 (PMC12811014; doi:10.1099/mic.0.001650)
Supplement: Uncited Supplementary Material 1. [file mic-172-01650-s001.pdf]

Phenylacetic acid mediates *Acinetobacter baumannii* entry into a viable but non-culturable state

Lyuboslava G Harkova, Rubén de Dios, Ronan R McCarthy

Supplementary Materials

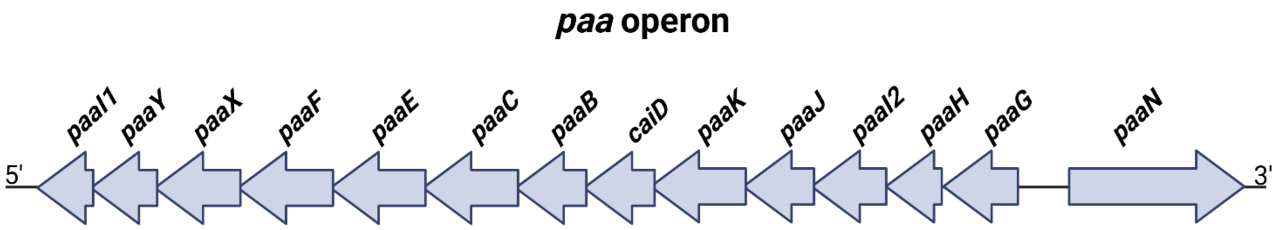

Supplementary Figure S1. Schematic representation of the *paa* operon in *A. baumannii* AB5075 (CP008706.1).

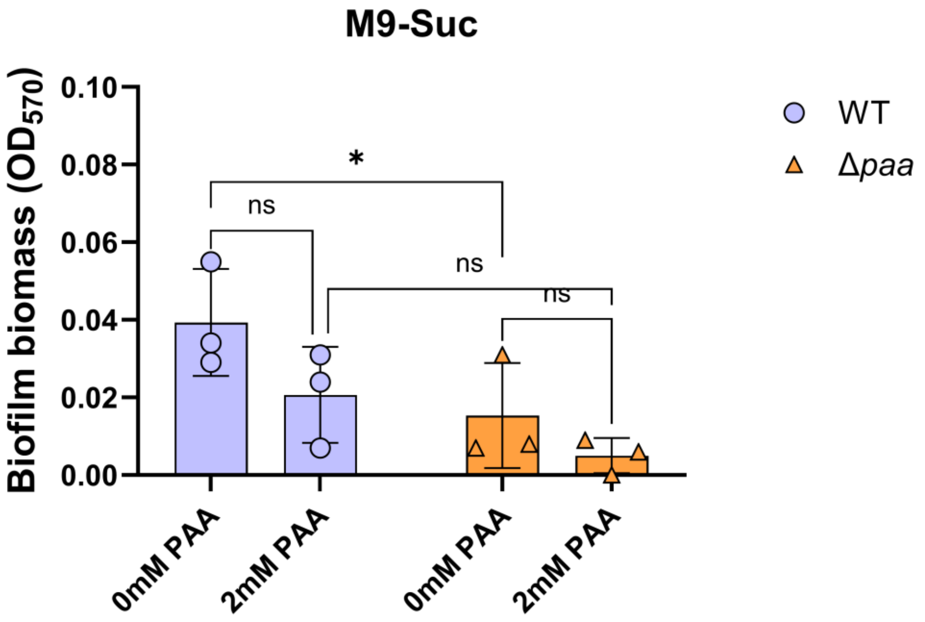

Supplementary Figure S2. Biofilm formation of AB5075 WT and Δ*paa* mutant after 24 h growth at 37 °C shaking in M9-succinate medium with 2 mM PAA and compared to controls without exogenous PAA. Biofilms were stained with 0.1% Crystal violet. The dye was resolubilised in 99% ethanol and absorbance was measured at OD_{570}.
